# Recent progress in quantum photonic chips for quantum communication and internet

**DOI:** 10.1038/s41377-023-01173-8

**Published:** 2023-07-14

**Authors:** Wei Luo, Lin Cao, Yuzhi Shi, Lingxiao Wan, Hui Zhang, Shuyi Li, Guanyu Chen, Yuan Li, Sijin Li, Yunxiang Wang, Shihai Sun, Muhammad Faeyz Karim, Hong Cai, Leong Chuan Kwek, Ai Qun Liu

**Affiliations:** 1 Quantum Science and Engineering Centre (QSec), Nanyang Technological University, Singapore, 639798 Singapore; 2 Institute of Precision Optical Engineering, School of Physics Science and Engineering, Tongji University, 200092 Shanghai, China; 3 School of Optoelectronic Science and Engineering, University of Electronic Science and Technology of China, 610054 Chengdu, China; 4 School of Electronics and Communication Engineering, Sun Yat-Sen University, 518100 Shenzhen, Guangdong China; 5 Institute of Microelectronics, A*STAR (Agency for Science, Technology and Research), Singapore, 138634 Singapore; 6 Centre for Quantum Technologies, National University of Singapore, 3 Science Drive 2, Singapore, 117543 Singapore; 7 National Institute of Education, Nanyang Technological University, Singapore, 637616 Singapore

**Keywords:** Quantum optics, Integrated optics

## Abstract

Recent years have witnessed significant progress in quantum communication and quantum internet with the emerging quantum photonic chips, whose characteristics of scalability, stability, and low cost, flourish and open up new possibilities in miniaturized footprints. Here, we provide an overview of the advances in quantum photonic chips for quantum communication, beginning with a summary of the prevalent photonic integrated fabrication platforms and key components for integrated quantum communication systems. We then discuss a range of quantum communication applications, such as quantum key distribution and quantum teleportation. Finally, the review culminates with a perspective on challenges towards high-performance chip-based quantum communication, as well as a glimpse into future opportunities for integrated quantum networks.

## Introduction

Quantum communication, which applies the principles of quantum mechanics for quantum information transmission, enables fundamental improvements to security, computing, sensing, and metrology. This realm encapsulates a vast variety of technologies and applications ranging from state-of-the-art laboratory experiments to commercial reality. The best-known example is quantum key distribution (QKD)^1,2^. The basic idea of QKD is to use the quantum states of photons to share secret keys between two distant parties. The quantum no-cloning theorem endows the two communicating users with the ability to detect any eavesdropper trying to gain knowledge of the key^3,4^. Since security is based on the laws of quantum physics rather than computational complexity, QKD is recognized as a desired solution to address the ever-increasing threat raised by emergent quantum computing hardware and algorithms.

Despite the controversy surrounding its practical security, QKD is leading the way to real-world applications^5^. For example, fiber-based and satellite-to-ground QKD experiments have been demonstrated over 800 km in ultra-low-loss optical fiber^6^ and 2000 km in free space^7^, respectively. The maximal secure key rate for a single channel has been pushed to more than 110 Mbit/s^8^. A number of field-test QKD networks have been established in Europe^9–11^, Japan^12^, China^13,14^, UK^15^, and so forth. Furthermore, the security of practical QKD systems was intensively studied to overcome the current technical limitations^5,16,17^. Post-quantum cryptography has been combined with QKD to achieve short-term security of authentication and long-term security of keys^18^.

Beyond QKD, quantum teleportation has attracted extensive attention, which exploits quantum entanglement for transferring fragile quantum information in an effectively unhackable manner^19–21^. Based on this, quantum networks can be conducted to connect various quantum devices, enabling unparalleled capabilities that are provably unattainable using only classical information techniques^22,23^. Quantum secure direct communication (QSDC)^24–26^, another important branch of quantum communication, has also provided opportunities for secure data transferring. This technique has been evolving quickly in recent years^27–33^, enabling users to directly transmit confidential information over secure quantum channels without sharing encryption keys. For instance, a QSDC network has been demonstrated with 15 clients^32^. Combined with post-quantum cryptography, a QSDC network with end-to-end security can be constructed using existing technologies^33^.

Conventional quantum communication systems are typically built using discrete optical devices. Generally, these devices are separately assembled with optical glasses (e.g., fused quartz and silica) and optical crystals (e.g., calcite, beta barium borate and lithium niobate) and connected via free space or optical fibers. Although it is convenient to optimize individual elements to fit with the strict requirements such as ultra-low loss, high efficiency, fast speed and high fidelity in quantum information applications, interconnects and packaging have always posed significant reliability and cost challenges for traditional discrete optical designs, especially when dealing with large-scale networks linking hundreds of thousands of users. For instance, high mechanical and thermal stabilities are required to mitigate space and phase misalignment over time due to environmental stresses and temperature variations, which are yet difficult to achieve in a complex discrete optical system by global stabilization. Therefore, current bulky systems composed of discrete optical components may struggle to meet the growing demand for higher volume transmission capability, manifesting great merits of chip-scale quantum communication systems^34^.

Quantum photonic chips are an ideal platform for new generation of quantum technology^35^. In addition to miniaturization, two advantages over discrete optical systems, i.e., scalability and stability are prominent. Scalability is enabled because the chips, with all their components, are printed as a unit by lithography rather than being constructed one component at a time. Stability is achieved as the circuits built on a robust and compact solid-state platform can minimize deviations due to vibrations or temperature variations. These two advantages are critical for achieving the level of integration and performance required for quantum information processing and highly efficient quantum communication. Moreover, quantum photonic chips have a strong potential for low-cost production. While the initial cost of fabricating the required photomasks is high, the average cost per chip can be greatly reduced through mass production.

After decades of effort, photonic integration has been realized in all aspects of individual quantum communication systems, including photon sources, encoding and decoding photonic circuits, and detectors^34,35^. In principle, integrated photonic chips can combine many desirable characteristics, such as efficiency, cost-effectiveness, scalability, flexibility and performance, that are required for quantum communication applications. Such characteristics, along with wafer-scale fabrication processes, make chip-based quantum communication systems a compelling platform for the future of quantum technologies.

In this review, we focus on the latest advances in implementing quantum communication on quantum photonic chips. We begin by discussing state-of-the-art integration platforms used for quantum photonics, summarizing their specific features and criteria that determine their suitability for quantum communication applications. Next, we examine the key elements of a chip-based quantum communication system, namely integrated photon sources, reconfigurable passive and active elements for manipulation of quantum states, and integrated single-photon and homodyne detectors. We then review progress in realizing on-chip systems for practical quantum communication implementations, including QKD and entanglement-based protocols such as entanglement distribution and quantum teleportation. Finally, we conclude by discussing the remaining challenges and prospects in this field.

## Key technologies for quantum photonic chips

**Fig. 1:**
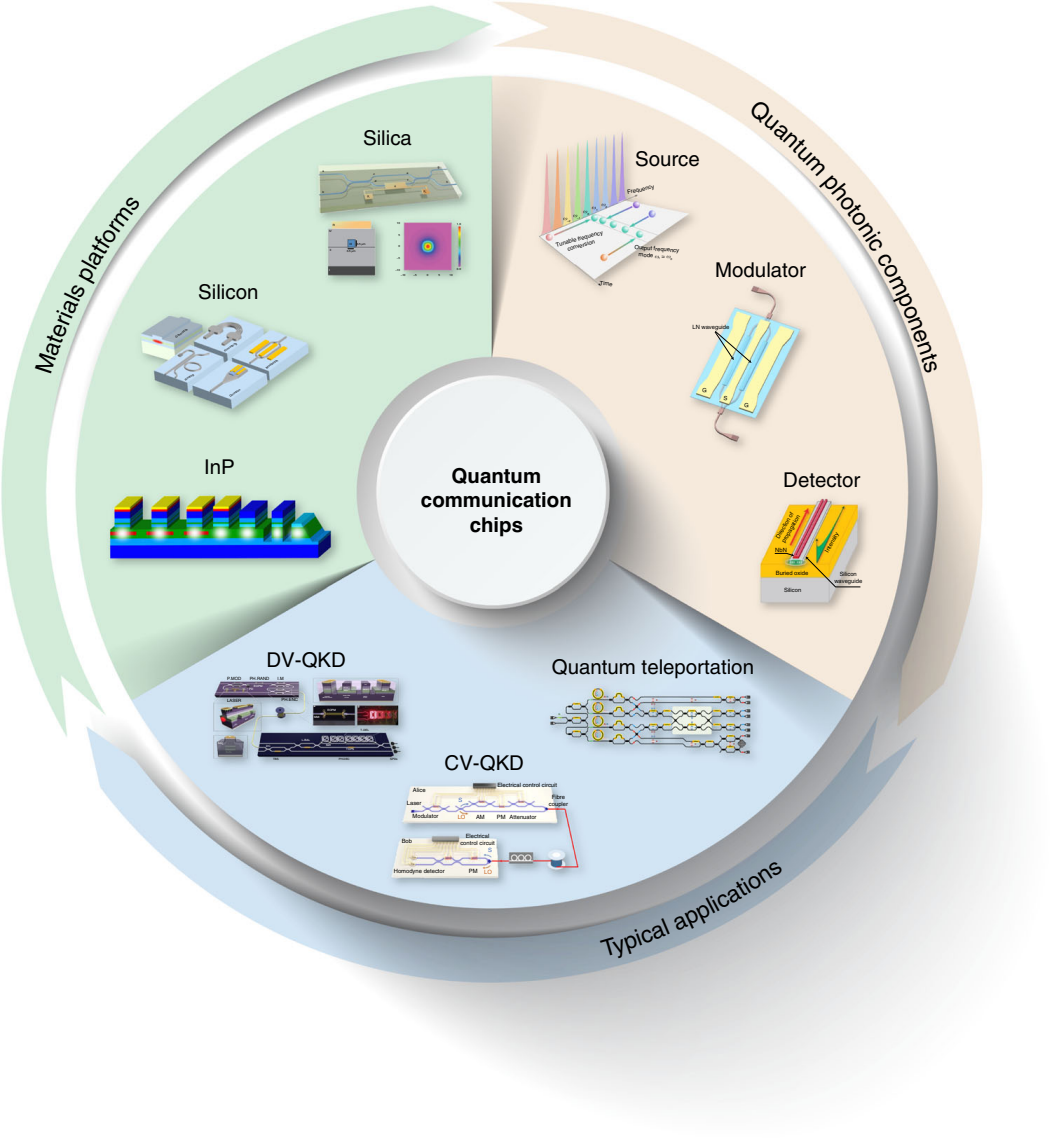
Overview of quantum photonic chips for quantum communication. The research scope covers photonic materials platforms for large-scale integration^36–38^, quantum photonic components such as quantum light sources^39^, high-speed modulators^40^, and highly efficient photodetectors^41^, as well as typical applications in QKD^42,43^ and quantum teleportation^44^. Panels reproduced with permission from: ref. ^36^, Springer Nature Ltd; ref. ^37^, under a Creative Commons licence (https://creativecommons.org/licenses/by/4.0/); ref. ^38^, AIP Publishing LLC; ref. ^39^, under a Creative Commons licence (https://creativecommons.org/licenses/by/4.0/); ref. ^40^, Springer Nature Ltd; ref. ^41^, under a Creative Commons licence (http://creativecommons.org/licenses/by-nc-nd/3.0/); ref. ^42^, under a Creative Commons licence (https://creativecommons.org/licenses/by/4.0/); ref. ^43^, Springer Nature Ltd; ref. ^44^, Springer Nature Ltd

Photonic integration opens the path towards miniaturized quantum communication systems with increasing complexity and enhanced functionality. Figure 1 provides an overview of the three aspects of integrated quantum communication: photonic materials platforms for large-scale integration^36–38^, quantum photonic components such as quantum light sources^39^, high-speed modulators^40^ and highly efficient photodetectors^41^, and typical applications in QKD^42,43^ and quantum teleportation^44^. Since the materials, preparation processes and structural designs employed in photonic integration are considerably different from those used in discrete systems, essential photonic components in chip-level configurations must be redesigned and optimized for specific quantum information applications. The relevant technical studies are summarized in this section, covering quantum light sources, encoding and decoding elements, quantum detectors and packaging techniques for integrated photonic systems.

**Fig. 2:**
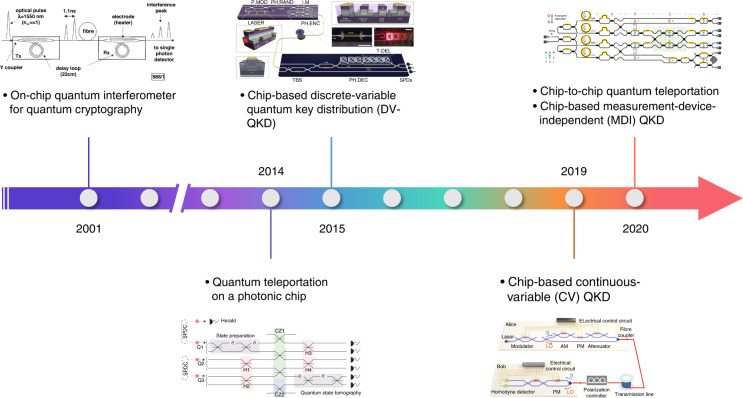
Timeline of advances in quantum photonic chips for quantum communication. The key milestones include the first demonstrations of the on-chip quantum interferometer for quantum cryptography^46^, quantum teleportation on a photonic chip^90^, chip-based DV-QKD^42^, CV-QKD^43^, and MDI-QKD^81,94,96^, and chip-to-chip quantum teleportation^44^. Panels reproduced with permission from: ref. ^46^, Institution of Electrical Engineers; ref. ^90^, Springer Nature Ltd; ref. ^42^, under a Creative Commons licence (https://creativecommons.org/licenses/by/4.0/); ref. ^43^, Springer Nature Ltd; ref. ^44^, Springer Nature Ltd

Figure 2 highlights key milestones in the development of integrated quantum communication. Early attempts in this field can be traced back to the integration of photon sources using periodically poled lithium niobate waveguides^45^ and interferometers using silica-on-silicon planar lightwave circuits (PLCs)^46–49^. The high efficiency and temperature-stabilized operation of these integrated elements demonstrated their inherent suitability over discrete, bulky components. Subsequently, many other materials were explored, and significant progress was made in on-chip generation, manipulation, and detection of quantum states of light for quantum communication and other quantum information applications.

Prevailing materials platforms for chip-based quantum communication implementations include silica waveguides (silica-on-silicon and laser-written silica waveguides), silicon-on-insulator (SOI), silicon nitride (Si_3_N_4_), lithium niobate (LN), gallium arsenide (GaAs), indium phosphide (InP) and silicon oxynitride (SiO_*x*_N_*y*_)^34,35,50^. Table 1 summarizes the state of the art of these monolithic platforms, indicating their advantages and disadvantages in terms of waveguiding properties, available active components, and compatibility with related technologies. For example, SOI provides a great refractive-index contrast for high-density integration, strong optical nonlinearity for nonclassical state generation, and excellent compatibility with advanced CMOS (complementary metal-oxide–semiconductor) processes that have been widely employed in the semiconductor industry. However, the lack of lasing capability makes it challenging to fully integrate all the required components of a quantum communication system. III–V semiconductor platforms (GaAs, InP, etc.) allow for monolithic system integration, yet coming at the expense of higher cost and lower integration level. The inevitable weaknesses of each material and its fabrication process indicate that no single platform can provide all the desired features for quantum communication applications. A viable solution is a hybrid integration that aims to combine the advantages of different platforms^50^. Such efforts have been made to realize heterogeneous quantum photonic devices like integrated superconducting nanowire single-photon detectors (SNSPDs)^41^ and integrated lasers for weak coherent pulse generation^51^. Other important technologies including semiconductor quantum dots (QDs) interfaced with photonic nanostructures^52^ and diamond-on-insulator^53,54^ have also emerged as competitive platforms for on-chip implementation of quantum communication.

**Table 1:** Specifications of the state of the art of monolithic integrated photonic platforms

	Silica waveguides^235^	Silicon-on-insulator^37,235^	Silicon nitride^236^	Lithium niobate on insulator^237^	Gallium arsenide^238^	Indium phosphide^38^	Silicon oxynitride^239,240^
Refractive index contrast	Low	High	Moderate	Moderate	Low	Low	Low, tunable
Losses	Ultralow	Moderate linear loss, High two-photon absorption	Low linear loss, low two-photon absorption	Moderate	Moderate	Moderate	Low linear loss, low two-photon absorption
Nonlinear index	Weak χ^3^	Strong χ^3^	Strong χ^3^	Strong χ^2^	Strong χ^2^	Strong χ^2^	Moderate χ^3^
Laser	N/A	N/A	N/A	N/A	Yes	Yes	N/A
Modulator	Low speed	High speed	Low speed	High speed	High speed	High speed	Low speed
Detector	N/A	Ge, High speed	N/A	N/A	High speed	High speed	N/A
Mode matching with optical fibers	Excellent	Poor	Moderate	Moderate	Poor	Poor	Moderate
CMOS compatibility	N/A	Excellent	Good	N/A	N/A	N/A	Good

*N/A* not applicable

### Quantum light sources

A photon source that generates designated quantum states of light is a key element of a quantum optical system. In general, single-photon states and entangled photon states are required in the architecture of quantum communication networks^16^, which can be obtained either deterministically using single-photon emitters or probabilistically using parametric nonlinear processes.

**Fig. 3:**
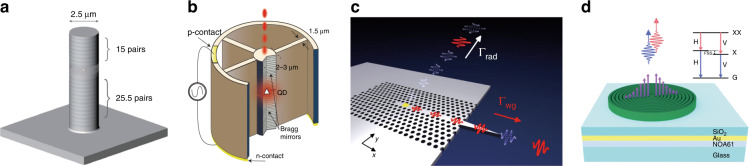
On-chip QD photon sources. **a** An illustration of a single InAs/GaAs self-assembled QD embedded in a 2.5-μm-diameter micropillar cavity^57^. **b** Schematic of an InGaAs QD coupled to a micropillar that is connected to a surrounding circular frame by four one-dimensional wires^58^. **c** A single QD embedded in a photonic crystal waveguide^59^. A large portion of emitted single photons is channeled with near-unity probability into the waveguide mode. **d** An illustration of a circular Bragg resonator on a highly efficient broadband reflector with a single GaAs QD emitting entangled photon pairs^64^. Panels reproduced with permission from: **a** ref. ^57^, APS; **b** ref. ^58^, Springer Nature Ltd; **c** ref. ^59^, APS; **d** ref. ^64^, Springer Nature Ltd

QDs are considered one of the most promising candidates for the on-demand generation of single photons or entangled photon pairs by virtue of the deterministic nature of their emission characteristics^55^. In particular, the small footprint and compatibility with semiconductor technology make them appealing for on-chip integration^56^. For single-photon generation, purity, extraction efficiency, and photon indistinguishability of 99.1%, 66%, 98.5% and 99.7%, 65%, 99.6% have been achieved in a single InAs/GaAs self-assembled QD (Fig. 3a)^57^ and an InGaAs QD (Fig. 3b)^58^, respectively. However, these micropillar-based QD single-photon sources present difficulty in waveguide integration due to their out-of-plane emission feature. Alternatively, QDs can be embedded in photonic crystal waveguides (Fig. 3c)^59^ or heterogeneous waveguide structures^60^ for highly efficient coupling with waveguides. Entangled photon pairs can also be obtained using the biexciton-exciton cascaded radiative processes in QDs^61–63^. By deterministically embedding GaAs QDs in broadband photonic nanostructures, an entangled photon pair source was demonstrated with a pair collection probability of 0.65, entanglement fidelity of 0.88, and indistinguishability of 0.901 and 0.903 (Fig. 3d)^64^. In addition to QDs, several other solid-state quantum emitters, such as color centers in diamond^53,54^, silicon carbide^65^, carbon nanotubes^66^, and defects in two-dimensional materials^67,68^, have also been investigated and shown great potential for on-chip generation of single photons or entangled photon pairs.

**Fig. 4:**
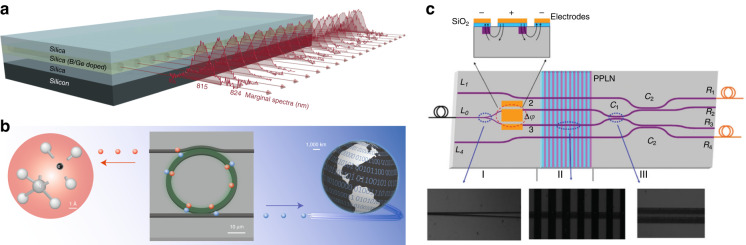
Different types of chip-based parametric photon sources. **a** Array of spontaneous four-wave mixing (SFWM) heralded single-photon sources (HSPSs)^70^. A series of straight waveguides are fabricated via UV-laser writing in a germanium-doped silica-on-silicon photonic chip, each of which constitutes its own HSPS. **b** A nanophotonic visible–telecom SFWM photon-pair source using high-quality factor silicon nitride resonators to generate narrow-band photon pairs with unprecedented purity and brightness^71^. **c** A spontaneous parametric down-conversion (SPDC) entangled photon source based on a LN photonic chip with a periodically poled section^72^. Panels reproduced with permission from: **a** ref. ^70^, under a Creative Commons licence (https://creativecommons.org/licenses/by/4.0/); **b** ref. ^71^, Springer Nature Ltd; **c** ref. ^72^, APS

Integrated probabilistic quantum light sources typically take advantage of spontaneous four-wave mixing (SFWM) or spontaneous parametric down-conversion (SPDC) in optical waveguides or other photonic structures (e.g., micro-disk and ring resonators, and photonic crystals). Due to the tight confinement of light, these nonlinear parametric processes are greatly enhanced on a chip, enabling efficient generation of high-quality photon states in miniaturized configurations. In SFWM, two pump photons are annihilated to produce a pair of signal and idler photons, where the frequencies of the pump (*ω*_p1_, *ω*_p2_), signal (*ω*_s_) and idler (*ω*_i_) must obey *ω*_p1_ + *ω*_p2_ = *ω*_s_ + *ω*_i_ to conserve the energy. Single-photon or entangled photon sources based on this four-photon process have been demonstrated in platforms with third-order nonlinearity, such as Si^69^, SiO_2_ (Fig. 4a)^70^, and Si_3_N_4_ (Fig. 4b)^71^. In SPDC, one pump photon is split into a pair of signal and idler photons, where the frequencies of the pump (*ω*_p_), signal (*ω*_s_), and idler (*ω*_i_) must also satisfy *ω*_p_ = *ω*_s_ + *ω*_i_. Photon sources based on this three-photon process have been implemented in platforms with second-order nonlinearity, such as periodically poled LN waveguide circuits (Fig. 4c)^72^ and a III–V semiconductor chip^73^. The major issues for these photon sources are that they produce photons non-deterministically and the generation rates are limited by the fundamental trade-off between brightness and multi-photon probability. Multiplexing techniques offer a promising way to solve the problems^39,74–77^. For instance, an integrated spatially multiplexed heralded single-photon source (HSPS) achieved 62.4% and 63.1% enhancement to the single photon generation probability for two separately pumped sources and two sources pumped through a common input, respectively^74^. Further improvement in efficiency requires better delay lines with ultra-low loss and miniaturized footprint, and faster switches with faster electronics to synchronize the operations^77^.

In a practical quantum communication system, single-photon sources and entangled photon sources are not always required. According to the decoy-state protocol^78–80^, weak coherent pulses can be used as a credible alternative to single-photon states for most prepare-and-measure QKD applications. As such, integrated photon sources can be achieved simply by attenuating the coherent pulses produced by on-chip lasers. Such photon sources have already been demonstrated in several chip-based QKD systems^42,51,81^.

### Reconfigurable quantum photonic components

**Fig. 5:**
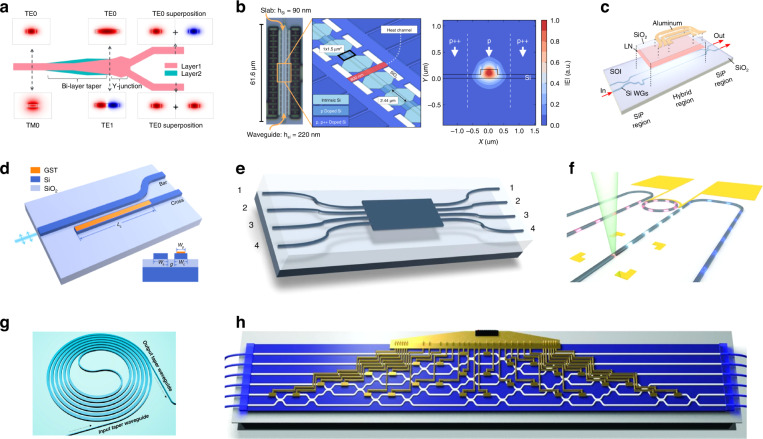
Typical integrated components on quantum photonic chips. **a** Schematics of a polarization splitter/rotator and the evolution of its mode profile^82^. **b** Optical micrograph and perspective view of a thermo-optic phase shifter in silicon^83^. **c** Schematic of a high-bandwidth electro-optic modulator, where an unpatterned LN thin film is bonded to a Mach-Zehnder interferometer fabricated in Si^84^. **d** Schematic of a directional coupler with a thin layer of Ge_2_Sb_2_Te_5_ (GST)^85^. **e** Schematic of a 4 × 4 multi-mode interferometer^86^. **f** Schematic of a hybrid quantum photonic circuit integrated with an on-chip tunable ring resonator filter^87^. **g** Schematic of a silicon photonic waveguide spiral delay line^88^. (h) A six-mode universal linear-optic device that was realized in a fully re-programmable silica chip^92^. Panels reproduced with permission from: **a** ref. ^82^, The Optical Society; **b** ref. ^83^, The Optical Society; **c** ref. ^84^, The Optical Society; **d** ref. ^85^, American Chemical Society; **e** ref. ^86^, under a Creative Commons licence (http://creativecommons.org/licenses/by-nc-nd/3.0/); **f** ref. ^87^, under a Creative Commons licence (https://creativecommons.org/licenses/by/4.0/); **g** ref. ^88^, Chinese Laser Press; **h** ref. ^92^, AAAS

Manipulation of quantum states of light is essential for the processing of quantum information in quantum communication, which can be readily implemented by using off-the-shelf passive and active components of integrated photonics. In a typical quantum communication system, photons are generally handled in polarization, phase, spatial, spectral, and temporal domains. Thus, it requires building blocks that can influence these degrees of freedom of the photons, such as polarization splitters/rotators (Fig. 5a)^82^, phase shifters (Fig. 5b)^83^, intensity modulators (Fig. 5c)^84^, directional couplers (Fig. 5d)^85^, multi-mode interferometers (MMI) (Fig. 5e)^86^, ring resonators (Fig. 5f)^87^, and delay lines (Fig. 5g)^88^. In particular, phase shifters can be realized via the thermo-optic effect for low-speed applications^83,89^ and the Pockels electro-optic effect for high-speed applications^40,84^. Such devices have been demonstrated in a variety of integrated platforms, e.g., an ultraviolet-written silica-on-silicon photonic chip for quantum teleportation with thermo-optic phase shifters^90^, a GaAs quantum photonic circuit with tunable Mach–Zehnder interferometer (MZI) relying on the Pockels effect^91^, a reprogrammable linear optical circuit comprising an array of 30 silica-on-silicon waveguide directional couplers with 30 thermo-optic phase shifters (Fig. 5h)^92^, and a large-scale silicon photonics quantum circuit integrating 16 SFWM photon-pair sources, 93 thermo-optical phase shifters and 122 MMI beam splitters^93^. On-chip modulators based on free-carrier dispersion effect^43,94^ or quantum-confined Stark effect^81^ can also be utilized for pulse generation and qubit encoding with frequencies up to GHz. For polarization-encoding protocols, modulators based on polarization rotators and polarization beam splitters have been designed and demonstrated for the generation of BB84 polarization states^94–96^.

Besides aforementioned elements, additional integrated components are required for optical connection between quantum photonic chips and optical fibers. One-dimensional grating couplers and off-plane coupling can be used when there is only one input or output polarization^97^. Otherwise, edge couplers like inverted tapers for butt coupling can be adopted instead in the case of more polarizations and wider spectral range^98^. Moreover, two-dimensional grating coupler supporting multi-polarization operation has been demonstrated to convert path-encoded qubits to polarization-encoded qubits that are more adapted for propagation in optical fibers^99,100^.

### Single-photon detectors (SPDs) and homodyne detectors

**Fig. 6:**
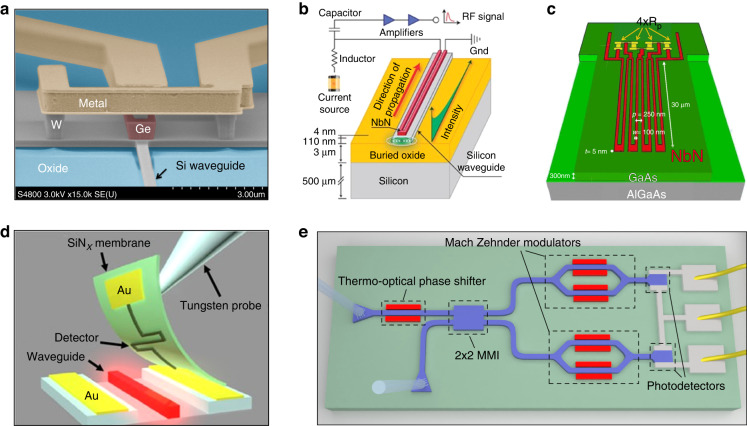
Overview of on-chip single-photon detector (SPD) and homodyne detector. **a** Angled scanning electron microscope image of a waveguide-coupled Ge-on-Si lateral single-photon avalanche photodiode with oxide cladding removed^101^. **b** A NbN nanowire traveling wave SNSPD atop a silicon waveguide with detection efficiency up to 91%^41^. **c** A waveguide photon-number resolving (PNR) SNSPD consisting of four wires in series with a resistance (*R*_p_) in parallel to each wire^106^. **d** Membrane transfer of a hairpin-shaped NbN SNSPD onto a photonic waveguide for on-chip detection of non-classical light^107^. **e** A silicon-based homodyne detector with a thermo-optical phase shifter, two Mach–Zehnder modulators and two photodiodes, interfaced to a customized transimpedance amplifier by wire bonding^114^. Panels reproduced with permission from: **a** ref. ^101^, The Optical Society; **b** ref. ^41^, under a Creative Commons licence (http://creativecommons.org/licenses/by-nc-nd/3.0/); **c** ref. ^106^, AIP Publishing LLC; **d** ref. ^107^, under a Creative Commons licence (https://creativecommons.org/licenses/by/4.0/); **e** ref. ^114^, The Optical Society

Efficient single-photon detection is of great importance to quantum communication applications. In particular, fully integrated SPDs are highly desirable because interfacing with off-chip detectors will lead to unavoidable coupling losses. Recently, an integrated waveguide-coupled Ge-on-Si lateral avalanche photodiode has been demonstrated for single-photon detection with efficiency of 5.27% at 1310 nm and a dark count rate of 534 kHz at 80 K (Fig. 6a)^101^. However, such single-photon avalanche photodiodes are often plagued with too many dark counts at high efficiencies. As an alternative, SNSPDs offer significantly lower dark noise with higher detection efficiency, reduced timing jitter, as well as photon-number resolving (PNR) capability. Waveguide-integrated SNSPDs have been reported in platforms of GaAs^102^, Si^41^, Si_3_N_4_^103,104^, LN^105^, etc., among which the traveling wave SNSPDs embedded on Si waveguides have achieved detection efficiency up to 91% and a dark count rate down to 50 Hz (Fig. 6b)^41^, and on-chip compatibility of reconfigurable components with SNSPDs was demonstrated at cryogenic temperatures^104,105^. Waveguide PNR detectors are possible by patterning multiple wires in series (Fig. 6c)^106^. In addition to direct deposition, large-scale integration of SNSPDs fabricated on silicon nitride membrane with silicon and aluminum nitride waveguides was enabled by using the pick-and-place technique (Fig. 6d)^107^. Moreover, transition-edge sensor (TES) detectors were implemented in a waveguide configuration for the PNR detection with a resolution of up to five photons^108,109^.

The balanced homodyne detector (or balanced zero-beat detector), which has been widely exploited in continuous-variable (CV) quantum information applications, is another crucial detection element for quantum measurement. Recent developments have significantly improved the performance of integrated homodyne detectors, enabling enhanced levels of compact size, good stability, broad bandwidth, low noise, and a high degree of common-mode rejection. As an illustration, a homodyne detector with 150-MHz bandwidth and 11-dB clearance was monolithically integrated onto a silicon photonics chip^110^. However, discrete amplification electronics greatly increase the device footprint. To reduce the size and total capacitance, wire bonding was utilized to integrate the germanium-on-silicon homodyne detector chip with the amplifier chip^111^, which resulted in a 3-dB bandwidth of 1.7 GHz and a shot-noise limited bandwidth of up to 9 GHz. A similar approach has also been applied to construct chip-level InGaAs homodyne detectors comprising low-parasitic photodiodes and low-noise high-speed transimpedance amplifiers^112,113^. Although it is convenient to adopt commercial telecom transimpedance amplifiers, they will typically bring suboptimal electrical noise. Co-design and integration of a homodyne detector with a customized transimpedance amplifier can efficiently reduce the noise and significantly boost the performance, allowing for a 20-GHz shot-noise-limited bandwidth and a quantum shot noise clearance of up to 28 dB (Fig. 6e)^114^.

### Chip packaging and system integration

While bare quantum photonic chips can be characterized using a probe station, they must be packaged into durable modules to develop working prototype devices^115^. To this end, numerous processes have been proposed to package quantum photonic chips into compact systems for real-world applications.

**Fig. 7:**
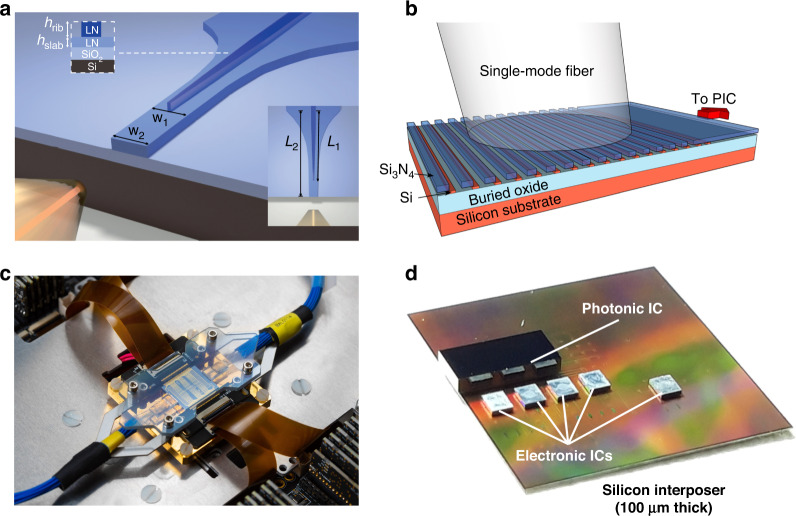
Instances of chip packaging and integration. **a** Schematic of a bilayer LN inverse taper coupled with a lensed optical fiber^121^. **b** Schematic of a Si_3_N_4_-on-SOI dual-level grating coupler interfaced with a single-mode fiber^123^. **c** Photograph of a quantum photonic processor packaged with PCBs, fiber arrays and thermoelectric cooler^127^. **d** Photograph of an assembled multi-chip module that provides connectivity between one photonic integrated circuit (IC) and four electronic ICs via silicon interposer^128^. Panels reproduced with permission from: **a** ref. ^121^, The Optical Society; **b** ref. ^123^, The Optical Society; **c** ref. ^127^, under a Creative Commons licence (https://creativecommons.org/licenses/by/4.0/); **d** ref. ^128^, under a Creative Commons licence (https://creativecommons.org/licenses/by/4.0/)

Generally, photonic packaging involves a range of techniques and technical competencies needed to make the optical, electrical, mechanical, and thermal connections between a photonic chip and the off-chip components in a photonic module^116–118^. Fiber-to-chip coupling is one of the best-known aspects. The main challenge associated with coupling between an optical fiber and a typical waveguide on the chip is the large difference between their mode‐field diameters (MFDs)^119^. For example, the MFD at 1550 nm is ~10 μm in telecom single‐mode fiber (SMF), while the cross-section of the corresponding strip silicon waveguide is usually only 220 × 450 nm. This mismatch can be mitigated by using configurations that efficiently extract the mode from waveguide^97^, such as inverted-taper edge couplers interfaced with lensed SMF fibers (Fig. 7a)^120,121^ or ultra-high numerical aperture fibers^122^, and grating couplers interfaced with SMF fibers (Fig. 7b)^119,123^. For the approach harnessing grating couplers, coupling efficiency up to 81.3% (−0.9 dB) can be achieved in a 260-nm-thick SOI platform without the need for a back reflector or overlayer^124^. Additionally, efficiencies over 90% have been experimentally demonstrated using edge couplers fabricated on 200-mm SOI wafers^125^. An alternative approach for cost-effective and panel-level packaging is the evanescent coupling scheme, which has been reported to have a coupling loss of approximately 1 dB at a wavelength of 1550 nm^126^.

To access the electrical components on quantum photonic chips, electronic packaging is required to route signals from electronic drivers, amplifiers, and other control circuitry. This is often achieved by interfacing with dedicated printed circuit boards (PCBs) (Fig. 7c)^127^. The connection between PCBs and the bond-pads on the chip is usually made using wire-bonds. When a very large number of electrical connections or precise sub-nanosecond control on multiple channels is needed, 2.5-dimensional or 3-dimensional integration with customized electronic integrated circuits (EICs) may be utilized (Fig. 7d)^115,128^. This integration can be achieved using either solder-ball-bump or copper-pillar-bump interconnects, providing a robust electrical, mechanical, and thermal interface for the photonic chips^129,130^.

Global thermal stabilization of quantum photonic devices is essential for prototypes that require high accuracy and repeatability or for field tests where seasonal temperature swings are common. This can be achieved using passive cooling techniques or a thermoelectric cooler (TEC). The added global stability from the TEC allows for more efficient and better reproducibility in the local temperature tuning of individual photonic elements (e.g., micro-ring resonators, thermo-optic phase shifters, etc.) on the chip^115^. Additionally, liquid cooling can be installed to further increase the cooling capacity of the system^127^.

## Quantum secure communication systems

As the most developed quantum secure communication technology, QKD based on bulk or fiber optic components has already been used in banks and governments to provide high-level security for data transmission. Nevertheless, wider applications require QKD systems that are more robust, compact, and can be mass manufactured at a lower cost. In the previous section, we have summarized a variety of studies targeting integrated devices for the realization of miniaturized and cost-effective quantum communication. In this section, recent efforts towards fully chip-based QKD platform are described from a system-level view. As an overview, the degree of integration for typical integrated QKD implementations is listed in Table 2. Further comprehensive reviews of the QKD protocols can be found in the refs. ^16,17,131,132^.

**Table 2:** Degree of integration for typical integrated QKD implementations

Reference	Platform	Protocol	QRNG	Source	Encoding	Decoding	Detector
Sibson, P. et al. (2017)^42^	InP, SiO_*x*_N_*y*_	BB84 COW DPS	No	Yes	Yes	Yes	No
Paraïso, T. K. et al. (2019)^153^	InP	DPS BB84	Yes	Yes	Yes	No	No
Paraïso, T. K. et al. (2021)^154^	InP, Si	Modified BB84	Yes	Yes	Yes	Yes	No
Ma, C. et al. (2016)^95^	Si	BB84	No	No	Yes	No	No
Sibson, P. et al. (2017)^155^	Si, SiO_*x*_N_*y*_	COW BB84	No	No	Yes	Yes	No
Bunandar, D. et al. (2018)^157^	Si	BB84	No	No	Yes	No	No
Avesani, M. et al. (2021)^158^	Si	BB84	No	No	Yes	No	No
Geng, W. et al. (2019)^159^	Si	BB84	No	No	Yes	Yes	No
Dai, J. et al. (2020)^160^	Si	COW DPS	No	No	Yes	Yes	No
Ding, Y. et al. (2017)^162^	Si	HD-QKD	No	No	Yes	Yes	No
Semenenko, H. et al. (2020)^81^	InP	MDI-QKD	No	Yes	Yes	No	No
Wei, K. et al. (2020)^94^	Si	MDI-QKD	No	No	Yes	No	No
Cao, L. et al. (2020)^96^	Si	MDI-QKD	No	No	Yes	Yes	No
Zheng, X. et al. (2021)^165^	Si, NbN	MDI-QKD	No	No	No	Yes	Yes
Zhang, G. et al. (2019)^43^	Si	CV-QKD	No	No	Yes	Yes	Yes

*COW* coherent one way, *DPS* differential phase shift, *HD-QKD* high-dimensional QKD

### Quantum random number generators (QRNGs)

The security of encryption is determined by the quality or unpredictability of keys, implying that a truly random number generator is an essential part of a quantum secure communication system. Although pseudo-random numbers are simple to create, their inherent deterministic behavior prevents them from being regarded as truly unpredictable. QRNGs have thus been developed to produce truly random numbers with characteristics of unpredictability, irreproducibility, and unbiasedness, which are guaranteed by the basic principle of quantum physics^133^.

**Fig. 8:**
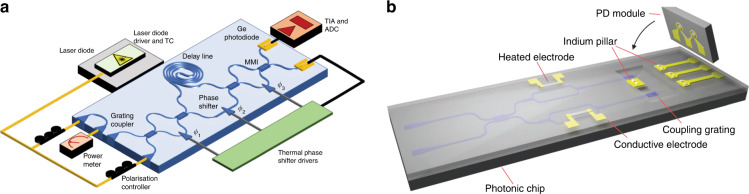
Integrated quantum random number generators (QRNGs). **a** A QRNG by measuring phase fluctuations from a laser diode with an SOI chip^146^. **b** A hybrid integrated QRNG with InGaAs photodiodes packaged on a SOI chip^112^. Panels reproduced with permission from: **a** ref. ^146^, Creative Commons licence (https://creativecommons.org/licenses/by/4.0/); **b** ref. ^112^, AIP Publishing LLC

The most commonly used protocols for QRNGs include the quantum phase fluctuation scheme^134–138^ and vacuum state scheme^139–144^. These schemes can easily achieve random bit rates up to Gbps by employing photodetectors instead of single-photon detectors. In addition to real-time output speed, the module size is also a key parameter of QRNG for practical applications. The emerging technology of integrated quantum photonics has exhibited considerable benefits in terms of size reduction. Recently, numerous integrated QRNG implementations have been demonstrated, leveraging various integration technologies with different levels of complexity. Utilizing multiplexed detectors, a QRNG based on LiNbO_3_ platform^144^ has reached a real-time rate of 3.08 Gbps, while a quantum entropy source has been constructed in an InP platform^145^. Since SOI platform has a higher integration density and superior technical maturity compared with III-V systems, QRNG implementations have also been reported on SOI platform by measuring phase fluctuations (Fig. 8a)^146^ and vacuum state^110^, respectively. However, it is worth noting that germanium photodiodes on SOI experience a significant dark current, which degrades the performance of on-chip QRNGs and needs careful optimization for mitigation. Alternatively, an integrated QRNG based on InGaAs photodiodes was constructed with a real-time output rate of 18.8 Gbps by virtue of a high bandwidth trans-impedance amplifier hybrid packaged with an SOI chip (Fig. 8b)^112^. Another integrated QRNG has been demonstrated based on a parallel array of independent single-photon avalanche diodes, homogeneously illuminated by a direct-current-biased light-emitting diode and co-integrated with logic circuits for postprocessing^147^. The real-time bit rate of the CMOS-based QRNG could reach up to 400 Mbps. Recently, through custom co-design of opto-electronic integrated circuits and side-information reduction by digital filtering, a record generation rate of 100 Gbps has been reported using an SOI photonic chip co-packaged with a GaAs transimpedance amplifier circuit^148^.

### DV-QKD systems

In typical QKD implementations, secret keys are encoded in discrete variables (DVs), such as the polarization or phase of photons. A prominent example of such DV-QKD protocols is decoy-state BB84^78–80^, which has been widely adopted in state-of-the-art commercial applications. According to the protocols, light sources, modulators, single-photon detectors, and essential passive optical components constitute the main framework of a DV-QKD system. Photonic integration of these elements began with the asymmetric PLC MZIs for differential-phase-shift QKD experiments^46–49^. The on-chip interferometers showed much more precise and stable operation for phase decoding compared to their fiber-based counterpart. Afterward, a series of compact QKD devices were demonstrated. For example, a miniaturized QKD transmitter was fabricated with a similar size to an electro-optic modulator, which incorporated a distributed feedback laser and a modulator^149^. The small-scale transmitter can produce 1550-nm weak-coherent pulses encoded in BB84 polarization states with decoy states. Then, a client consisting of an on-chip LiNbO_3_ polarization rotator was realized for client-server reference-frame-independent QKD^150^. The client integrated into a handheld device received dim laser pulses from a QKD server, and then attenuated and encoded each pulse with a qubit of information for return transmission to the server. In addition, the design and evaluation of a handheld QKD transmitter module were put forward based on an integrated optics architecture with an effective size of 25 mm × 2 mm × 1 mm^151^. In the module, four vertical-cavity surface-emitting lasers coupled to four micro-polarizers fabricated by focused ion beam milling were used to generate polarization qubits. The qubits were combined with a waveguide array fabricated in borosilicate glass for ensuring spatial overlap.

**Fig. 9:**
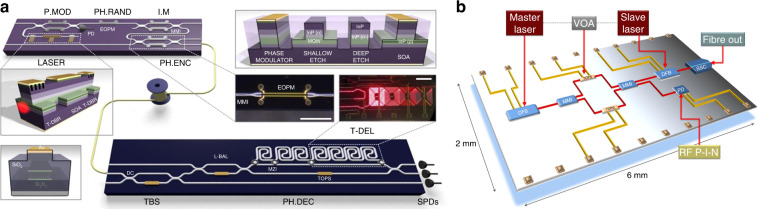
Chip-based QKD systems with hybrid materials platform. **a** A chip-to-chip system with a 2 × 6 mm^2^ integrated indium phosphide (InP) transmitter and a 2 × 32 mm^2^ silicon oxynitride (SiO_*x*_N_*y*_) photonic receiver circuit for GHz clock rate, reconfigurable, multi-protocol QKD^42^. **b** A modulator-free QKD transmitter chip consisting of two cascaded high-bandwidth distributed feedback lasers and one variable optical attenuator^153^. Panels reproduced with permission from: **a** ref. ^42^, under a Creative Commons licence (https://creativecommons.org/licenses/by/4.0/); **b** ref. ^153^, under a Creative Commons licence (https://creativecommons.org/licenses/by/4.0/)

The devices previously discussed show the viability and feasibility of partially integrated QKD systems. Nevertheless, fully chip-based systems are essential for enhanced performance, miniaturization, and increased functionality necessary in practical deployments. A QKD system was demonstrated with a high degree of integration (Fig. 9a)^42^. The transmitter module on an InP chip and the receiver module on a SiO_*x*_N_*y*_ chip was integrated by using components and manufacturing processes from the telecommunication industry. The InP transmitter monolithically incorporated a tunable laser, optical interferometers, electro-optic phase modulators and a p–i–n photodiode, while the SiO_*x*_N_*y*_ receiver consisted of thermo-optic phase shifters and a reconfigurable delay line that interfaced with off-chip single photon detectors. The reconfigurability of the devices enabled the implementation of multiple protocols, including BB84, coherent one-way, and differential phase shift, with clock rates up to 1.7 GHz, a quantum bit error rate (QBER) as low as 0.88%, and estimated secret key rates up to 568 kbps for an emulated 20 km fiber link. Recently, the data rate of the chip-based system has been increased through wavelength division multiplexing (WDM)^152^. Such WDM-QKD system was implemented using two InP transmitters and a single SiO_*x*_N_*y*_ receiver with on-chip asymmetric MZI filters for wavelength demultiplexing. The combined WDM channels doubled the secret key rate to 1.11 Mbit/s over a 20 km emulated fiber. The aforementioned implementations of chip-based QKD systems relied on integrated modulators. In fact, a modulator-free QKD transmitter chip can be realized based on the direct phase modulation approach recently introduced in bulk optics transmitters. Using the modulator-free chip, secure key rates of 270 and 400 kbps at 20 dB attenuation were achieved for the decoy state BB84 and distributed phase shift protocols, respectively (Fig. 9b)^153^. Recently, an entirely standalone QKD system has been developed based on InP photonic integrated circuits assembled into compact modules^154^. This system integrates the quantum transmitter, receiver, and QRNG chips, enabling quantum random number generation and key distribution at gigahertz clock rates.

**Fig. 10:**
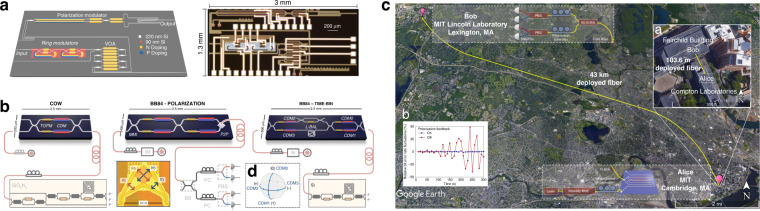
Silicon photonic chips for multiple QKD protocols. **a** A Si transmitter for polarization-encoded QKD, consisting of a microring pulse generator, a microring intensity modulator, four variable optical attenuators, and a polarization controller^95^. **b** Integrated silicon photonic transmitters to perform multiple QKD protocols, including coherent-one-way, polarization encoded BB84 and time-bin encoded BB84^155^. **c** Field test of intercity QKD over a 43-km dark fiber link using a silicon photonic polarization encoder^157^. Panels reproduced with permission from: **a** ref. ^95^, The Optical Society; **b** ref. ^155^, under a Creative Commons licence (https://creativecommons.org/licenses/by/4.0/); **c** ref. ^157^, under a Creative Commons licence (https://creativecommons.org/licenses/by/4.0/)

Silicon photonics is another attractive platform suitable for fully chip-based QKD systems. Although integrating light sources and SPDs remains challenging, several proof-of-principle demonstrations of Si-based QKD devices have been reported in recent years. One early work showcased a Si optical transmitter for polarization-encoded QKD (Fig. 10a)^95^. The chip incorporated a pulse generator, intensity modulator, variable optical attenuator, and polarization modulator in a 1.3 mm × 3 mm die area and executed the BB84 protocol with a QBER of 5.4% and an asymptotic secure key rate of 0.95 kbps over a 5-km fiber link. Meanwhile, three implementations of high-speed low-error QKD with silicon photonic devices were demonstrated (Fig. 10b)^155^. Employing a combination of thermo-optic phase modulators alongside high-bandwidth carrier-depletion modulators, they attained estimated asymptotic secret key rates of up to 916 kbps and QBERs as low as 1.01% over 20 km of fiber. Furthermore, a silicon photonic transceiver circuit was constructed, capable of generating the four BB84 states with >30 dB polarization extinction ratios and gigabit-per-second modulation speed^156^. On this basis, polarization-encoded QKD field tests were demonstrated using a similar silicon photonic encoder (Fig. 10c)^157^. The systems achieved composable secret key rates of 1.039 Mbps in a local test (on a 103.6-m fiber with a total emulated loss of 9.2 dB) and 157 kbps in an intercity metropolitan test (on a 43-km fiber with 16.4 dB loss). In addition, other demonstrations using silicon photonics have also been reported, including an integrated state encoder for free-space daylight QKD^158^, a silicon photonic QKD transceiver based on time-bin protocol^159^, a silicon photonic transmitter for high-speed distributed-phase-reference QKD^160^ and an integrated QKD receiver for multiple users^161^.

**Fig. 11:**
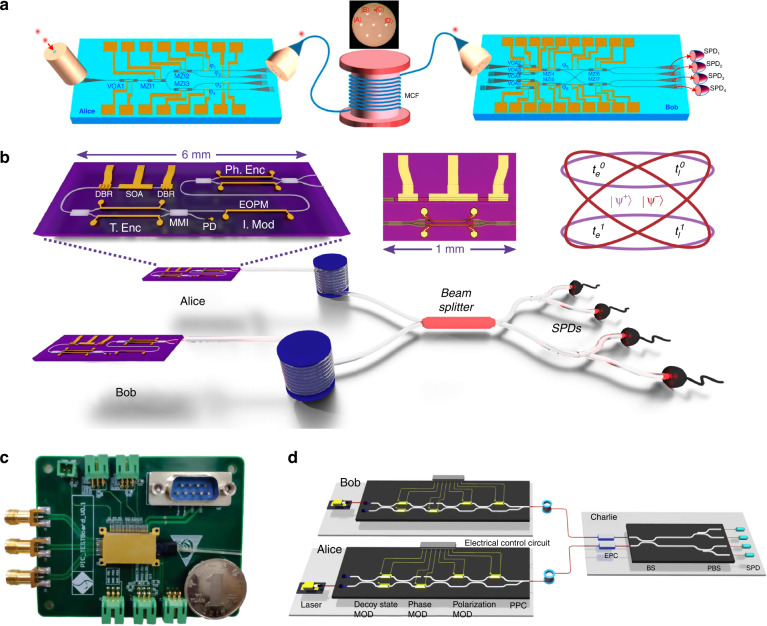
Different chip-based quantum communication systems for advanced QKD protocols. **a** Silicon-photonic-integrated circuit for noise-tolerant high-dimensional QKD^162^. **b** InP transmitter chips used to generate the time-bin encoded BB84 weak coherent states for MDI-QKD^81^. **c** A packaged silicon photonic MDI-QKD transmitter chip soldered to a compact control board^94^. **d** A silicon photonic chip-based MDI-QKD system comprising two transmitter chips and one server chip interfaced with off-chip SPDs^96^. Panels reproduced with permission from: **a** ref. ^162^, under a Creative Commons licence (https://creativecommons.org/licenses/by/4.0/); **b** ref. ^81^, under a Creative Commons licence (https://creativecommons.org/licenses/by/4.0/); **c** ref. ^94^, under a Creative Commons licence (https://creativecommons.org/licenses/by/4.0/); **d** ref. ^96^, APS

Recently, implementations of advanced QKD protocols with chip-based systems have garnered more interest, as these protocols would greatly benefit from photonic integration. A noise-tolerant high-dimensional QKD protocol based on space division multiplexing in multicore fiber was demonstrated using silicon photonic integrated circuits (Fig. 11a)^162^. These circuits provided a much more efficient way to create high-dimensional quantum states, enabling low and stable QBER well below both the coherent attack and individual attack limits. Moreover, measurement-device-independent (MDI) QKD, which eliminates all side channel loopholes in detection, is well-suited for a chip-based client-server scenario, where clients hold low-cost photonic chips, and the server, acting as an untrusted node, incorporates the most expensive elements that can be shared among multiple users. The feasibility of using integrated photonics for MDI-QKD was demonstrated in two independent studies with the InP platform^163^ and the Si/III–V hybrid platform^51^, respectively. In these studies, Hong–Ou–Mandel interference, the key component of MDI-QKD, was performed between weak coherent states from the chips. High visibilities of 46.5 ± 0.8% and 46 ± 2% were observed with two InP transmitters^163^ and two III–V on silicon waveguide integrated lasers^51^, respectively. Subsequently, five research groups implemented chip-based MDI-QKD systems: a star-topology quantum access network with an integrated server was built for MDI-QKD^164^; secure key exchange up to 200 km was presented using monolithically integrated InP transmitters (Fig. 11b)^81^; a 1.25-GHz MDI-QKD system was reported with two silicon photonic transmitters (Fig. 11c)^94^; an all-chip-based MDI-QKD system including two client chips and one server chip was demonstrated using silicon photonics (Fig. 11d)^96^; and a fully integrated relay server for MDI-QKD was realized based on a heterogeneous superconducting-silicon-photonic chip^165^.

### CV-QKD systems

**Fig. 12:**
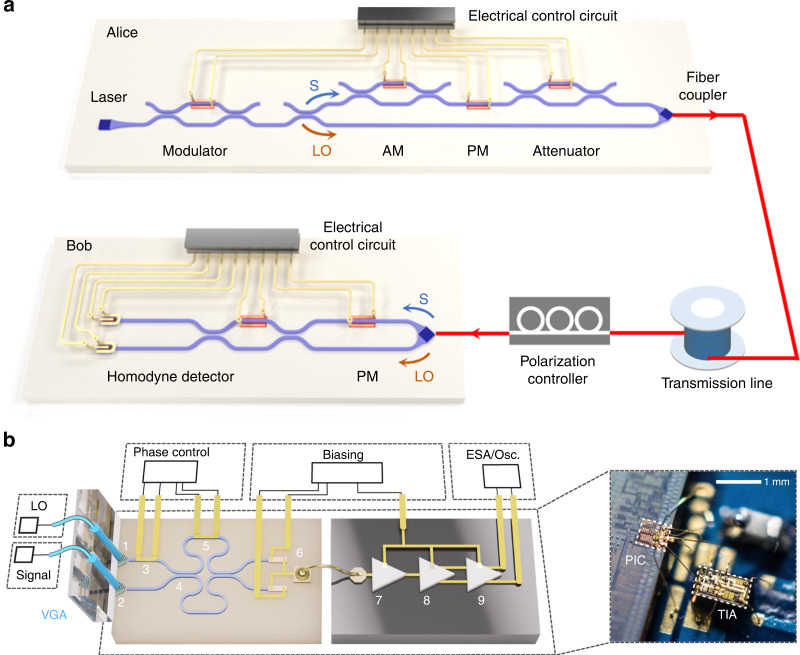
Integrated circuits for continuous variable (CV) QKD and high-speed homodyne detection. **a** An integrated silicon photonic chip platform for CV-QKD, consisting of a transmitter for signal modulation and multiplexing and a receiver for signal demultiplexing and homodyne detection^43^. **b** A silicon photonic homodyne detector interfaced with integrated electronics for 9-GHz measurement of squeezed light^111^. Panels reproduced with permission from: **a** ref. ^43^, Springer Nature Ltd; **b** ref. ^111^, Springer Nature Ltd

In addition to DV-QKD, several QKD protocols^166–168^ have been proposed to encode key information into continuous variables, such as the values of the quadrature components of the quantized electromagnetic field. A major technical difference is that CV-QKD implementation requires only homodyne detectors, rather than the dedicated SPDs used in DV-QKD. This feature eliminates the need for an additional cryogenic system and dramatically simplifies the detection setup. Consequently, CV-QKD is naturally suitable for photonic integration and compatible with chip-based coherent detection schemes that have been used in classical high-bandwidth communication systems. Indeed, a silicon photonic transceiver design was proposed comprising all major CV-QKD components as well as complete subsystems^169^; the feasibility of a homodyne detector integrated onto a photonic chip was demonstrated for measuring quantum states and generating random numbers^110^. Recently, a stable and miniaturized system was implemented for CV-QKD, compatible with existing fiber communication infrastructure by integrating all optical components (except the laser source) on a silicon photonic chip (Fig. 12a)^43^. The proof-of-principle characterization demonstrated that the system was capable of producing a secret key rate of 0.14 kbps (under collective attack) over a simulated distance of 100 km in fiber. The performance of chip-based CV-QKD systems can be improved by further optimizing the detection module. As a possible illustration, a high-speed homodyne detector was realized by interfacing CMOS-compatible silicon and germanium-on-silicon nanophotonics with silicon-germanium integrated amplification electronics (Fig. 12b)^111^. The detector has a 3-dB bandwidth of 1.7 GHz, a shot-noise limited to 9 GHz, and requires only a miniaturized footprint of 0.84 mm^2^.

## Entanglement distribution and quantum teleportation systems

Quantum teleportation has been demonstrated with many platforms ranging from superconducting qubits, trapped atoms, nitrogen-vacancy centers, to continuous variable states and so forth^170^. Among these implementations, photonic qubit is one of the most promising candidates to build the quantum channel in a quantum network since it is robust in a noisy environment and easy to manipulate at room temperature^23^. Moreover, it can tolerate longer propagation distances with minimal disturbance from the surroundings. So far, photonic quantum teleportation has been implemented experimentally in many ways including free space and fiber systems^170^.

When quantum teleportation was first experimentally verified, qubits were encoded in the polarization of photons generated from a BBO crystal in a free-space system on an optical table^20^. Later, the record for free-space teleportation was pushed up to over 1400 km between the Micius satellite and a ground station^171^. This achievement paved the way for an interconnected quantum network globally. However, considering the challenges of beam divergence, pointing, and collection for free-space teleportation, optical fiber systems are more promising for cost-effective metropolitan quantum networks. Currently, the longest fiber-based teleportation distance achieved is 102 km^172^.

One of the main challenges in photonic qubit teleportation is that the theoretical efficiency of Bell state measurement is limited to only 50% when using linear optics. To overcome this limitation, the continuous variable optical mode can be adopted as an alternative for realizing fully deterministic state teleportation. This approach has already been demonstrated over a 6-km fiber channel^173^. However, its fidelity still needs to be improved, as this scheme is sensitive to channel loss. For other types of material qubits, a record distance of 21 m has been achieved using trapped atom systems^174^.

As quantum teleportation continues to make strides toward real-world applications, the importance of integration as a key technology has become increasingly evident. In a future quantum network, it will be possible to embed a teleportation chip into stationary hardware (e.g., relays in the station) or mobile hardware (e.g., drones^175^) to transform these devices into lightweight and compact quantum nodes. This would enable remote access to quantum equipment for sharing quantum information or unlocking greater computational power. Such advancements have been made possible owing to the ability to generate and manipulate entangled photon pairs in different degrees of freedom on chip^176^, such as the path-encoded entangled states in MZIs^93^, polarization-encoded entangled states by engineering birefringent structures^177^, and time-bin entangled states in Franson interferometers^178^.

**Fig. 13:**
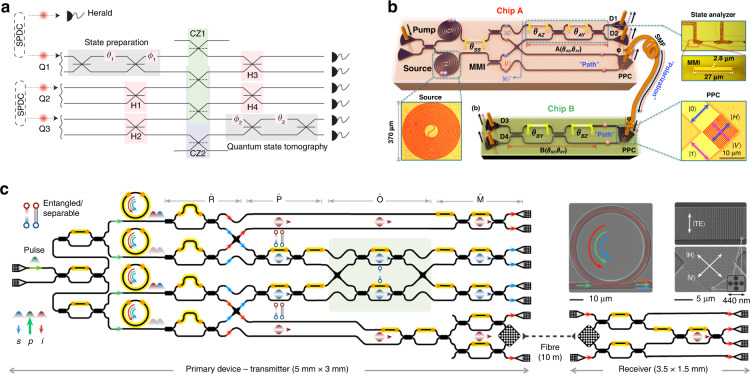
Chip-based quantum teleportation and entanglement distribution systems. **a** Scheme of an on-chip quantum teleportation experiment in a silica-on-silicon integrated chip^90^. **b** Silicon photonic circuit diagram for a chip-to-chip entanglement distribution experiment^100^. **c** Photonic circuit diagram for a chip-to-chip quantum teleportation experiment^44^. Panels reproduced with permission from: **a** ref. ^90^, Springer Nature Ltd; **b** ref. ^100^, The Optical Society; **c** ref. ^44^, Springer Nature Ltd

The first on-chip teleportation (Fig. 13a) was reported with off-chip photon source and achieved a fidelity of 0.89, although it was performed within a single chip^90^. Recent technological progress in integrated quantum photonics has enabled the implementation of entanglement-based quantum communication protocols beyond a single chip. The first chip-to-chip entanglement distribution was demonstrated with all key components monolithically integrated on silicon photonic chips (Fig. 13b)^100^. On-chip entangled Bell states were generated, and one qubit was distributed to another silicon chip by converting on-chip path-encoded states and in-fiber polarization states via the two-dimensional grating couplers. Furthermore, more integrated quantum circuits with on-chip sources have realized inter-chip teleportation with a fidelity of 0.88 (Fig. 13c)^44^. This chip-scale demonstration of photonic qubit production, processing, and transmission shows a promising way for the distributed quantum information processing internet. Moreover, entangled photon pairs across the visible-telecom range were demonstrated on a Si_3_N_4_ chip with a delicately engineered micro-ring resonator and further distributed over 20 km^71^. High photon number purity and brightness were achieved with low pump consumption of hundreds of microwatts. Importantly, it provides an entangling link between visible-band photons that can interface with quantum memories and telecom-band photons that feature low-loss transmission in optical fibers.

## Challenges and perspectives

In this review, the rapid advances in chip-based quantum communication relying on the development of integrated quantum photonics are discussed. Photonic integration not only provides a solid strategy for the miniaturization and scaling of quantum communication systems but also fosters practical applications of quantum communication and paves the way for future quantum communication networks and the quantum internet.

Although considerable progress has been achieved, the field of chip-based quantum communication is still in its early stages and naturally faces many challenges. On the component side, on-chip elements used in quantum communication require more stringent specifications than those used in classical optical communication to ensure high fidelity and prevent decoherence of quantum states during the process of preparation, manipulation, transmission, and detection. Hence, the exploration of components with suitable characteristics is crucial. For example, high-key-rate QKD calls for modulators that can operate at high clock rates while maintaining an acceptable extinction ratio for low crosstalk between different quantum states. However, this demand cannot always be satisfied by conventional Si-based modulators because carrier injection or carrier depletion techniques induce non-ideal loss characteristics. Fortunately, recent progress in ultra-high extinction (>65 dB) Si modulators based on a cascaded MZI structure^179^ and LN^180^, Si-LN^40^, and Si-barium titanate^181^ modulators based on the electro-optical Pockels effect provides possible solutions to this problem.

On the system side, fully integrated quantum communication systems with photon sources, photonic circuits, and detectors have not yet been realized. The difficulties in achieving full integration are due to two challenges: (i) the first challenge is that no monolithic platform can provide all the desired features for quantum communication applications. Hybrid integration, as discussed in the section “Key technologies for quantum photonic chips”, could be a viable solution to address this problem. However, the technique is still under development and requires more effort to achieve the final goal. Fortunately, a detailed roadmap for realizing future large-scale hybrid integrated quantum photonic systems has been summarized^50^; (ii) the second challenge is that different parts of an integrated quantum system may work in different conditions. For example, QD single-photon sources and single-photon detectors usually operate at cryogenic temperatures. In contrast, conventional integrated modulators and thermo-optic phase shifters are designed for room temperature applications and cannot function properly under these extreme conditions. Photon manipulation at cryogenic temperatures has thus become a crucial factor for fully integrated systems. Recently, an integrated cryogenic Si-barium titanate modulator^182^ and microelectromechanical photonic circuits interfaced with SNSPDs on the same chip^104^ have been demonstrated, removing major roadblocks for the realization of cryogenic-compatible systems. Furthermore, at a practical level, targeting truly useful systems with potential for industrial development will require the integration of both optics and electronics. A recent demonstration has shown the feasibility of integrating photonics with silicon nanoelectronics to construct complete systems on a chip for quantum communication^183^.

On the security side, chip-based quantum communication faces potential loophole threats due to the specific imperfections of integrated photonic devices. For instance, phase- and polarization-dependent losses are significant problems in quantum photonic chips that, if unchecked, could lead to an overestimation of the secret key rate, compromising the security of QKD systems. To solve these problems, a post-selection scheme has recently been proposed that provides a high key generation rate even in the presence of severe phase- and polarization-dependent losses^184^. A decoy-state BB84 QKD experiment considering polarization-dependent loss exploited the proposal and successfully distributed secure key bits over fiber links up to 75 km^185^. Additionally, the security loopholes originating from the plasma dispersion effect of free carriers^186^ and the integrated electrical control circuit of the transmitter^187^ have been revealed and analyzed in chip-based CV-QKD systems. Since there are still doubts about practical QKD implementations from government organizations like the National Security Agency (NSA) of the USA and the National Cyber Security Centre (NCSC) of the UK, further studies with comprehensive security analysis are needed to close the gap between theoretical models and practical integrated quantum communication systems.

Beyond prepare-and-measure QKD, entanglement-based QKD is another promising application for future chip-based QKD systems. This has become possible since time-bin entangled states were generated in GaAs^188^, Si^189^ and Si_3_N_4_^71,178^ chips, and the chip-to-chip entanglement distribution^100^ and quantum teleportation^44^ were demonstrated between two programmable Si chips. Combined with recent experimental progress^190–192^, integrated photonics provide a viable way for the realization of compact entanglement-based systems that support device-independent QKD over kilometer-scale distances. In addition, QSDC can also utilize the great potential of quantum photonic chips in developing practical QSDC systems and networks as the protocol share a similar setup with QKD^27,31,32^.

Currently, on-chip quantum teleportation is mostly based on posterior and passive protocols. Future work may include implementing feed-forward control by upgrading a quantum communication system from passive to active so that the receiver can apply conditional unitary operation in real-time to reconstruct quantum states. Furthermore, long-distance entanglement distribution and quantum teleportation and large-scale implementations of quantum networks rely on quantum memories and quantum relays^22,193^. For example, quantum memories in quantum nodes can generate entanglement between distant parties and therefore extend the communication distance. However, the experimental development of integrated quantum memory is still in its infancy. There is still much work to be done to achieve integrated quantum relays in the telecom band that are compatible with fiber-based long-distance quantum communication systems.

For practical applications of quantum communication, the loss and decoherence in transferring photons between different chips, either through optical fiber or free space, can greatly limit the fidelity of the network. Several solutions have been proposed to address the problem originating from optical coupling. For instance, edge coupling via a tapered silicon waveguide surrounded by a SiO_2_ cladding cantilever structure was utilized, shrinking the coupling loss to 1.3 dB/facet^194,195^. By engineering the effective refractive index of the waveguides, subwavelength waveguide grating-based edge couplings could achieve coupling efficiencies as high as 0.32 dB/facet (93%)^196^. For the packaging process of multiport coupling, methods using intermediate mode transformation stages waveguide such as ribbon layers^197^, photonic wire bonding^198^, and 3D printing free-form lenses and mirrors^199^ were employed to reduce coupling loss. However, the edge coupling strategy is frequently constrained by the effective modal refractive index and mode-size mismatch between the waveguide and fiber due to fabrication or alignment deviation. The technique using evanescent coupling between tapered waveguides and single-sided conical tapered fibers could overcome this shortage and produce highly effective coupling up to 0.13 dB/facet (97%)^200^.

As another factor that drives the compact integration of optical components, quantum computing on integrated photonic chips has also attracted much attention in recent years. There are two types of optical models^201^: specific quantum computing models^202,203^ (e.g., boson sampling), and universal quantum computing models^204–209^ (e.g., one-way or measurement-based). For specific quantum computation, a variety of photonic systems were demonstrated using quantum photonic chips^210–217^, enabling a natural and effective implementation of boson sampling. Gaussian boson sampling^218,219^, which can dramatically enhance the sampling rate with the adoption of squeezed light sources, was performed for the calculation of molecular vibronic spectra on a Si chip^217^ (up to 8 photons) and a SiN chip^216^ (up to 18 photons). Recently, quantum computational advantage has been delivered by photonic Gaussian boson sampling processors^220,221^, paving the path for further development of integrated specific quantum computers with potential applications including graph optimization^222^, complex molecular spectra^223^, molecular docking^224^, quantum chemistry^225^, etc. For universal quantum computation, a number of major functionalities have been demonstrated with on-chip photonic components, such as controlled-NOT gate and its heralding version^92,226^, and compiled Shor’s factorization^227^. Moreover, both architectural and technological efforts have been dedicated to photonic one-way quantum computation. This approach employs cluster states and sequential single-qubit measurement to perform universal quantum algorithms^205,207,228^ and can be greatly enhanced by implementing resource state generation and fusion operation natively^229–231^. The relevant circuit implementations include programmable four-photon graph states on a Si chip^232^, path-polarization hyperentangled and cluster states on a SiO_2_ chip^233^ and programmable eight-qubit graph states on a Si chip^234^.

In conclusion, quantum photonic chips have rapidly matured to become a versatile platform that proves to be invaluable in the development of cutting-edge quantum communication technologies. This review delves into the advancements achieved in this particular field. Considering the remarkable outcomes, it is anticipated that photonic integration will eventually assume a crucial role in building various quantum networks and potentially a global quantum internet, reshaping the landscape of future communication methodologies.
